# Social disparities in the use of colonoscopy by primary care physicians in Ontario

**DOI:** 10.1186/1471-230X-11-102

**Published:** 2011-09-28

**Authors:** Binu J Jacob, Nancy N Baxter, Rahim Moineddin, Rinku Sutradhar, Lisa Del Giudice, David R Urbach

**Affiliations:** 1 Clinical Decision Making & Health Care, Toronto General Hospital, 200 Elizabeth Street, Toronto, Ontario, M5G 2C4, Canada; 2 Institute for Clinical Evaluative Sciences, 2075 Bayview Avenue, Toronto, Ontario, M4N 3M5, Canada; 3 Department of Surgery and Keenan Research Centre, Li Ka Shing Knowledge Institute, St. Michael's Hospital, 30 Bond Street, Toronto, Ontario, M5B 1W8, Canada; 4 Department of Health Policy Management Evaluations, University of Toronto, Health Sciences Building155 College Street, Suite 425, Toronto, ON M5T 3M6, Canada; 5 Department of Family and Community Medicine, University of Toronto, 263 McCaul Street, Toronto, Ontario, M5T 1W7, Canada; 6 Dalla Lana School of Public Health, University of Toronto, 155 College Street, Toronto, Ontario, M5T 3M7, Canada; 7 Sunnybrook Health Science Centre, Bayview Avenue, Toronto, Ontario, M4N 3M5, Canada; 8 Department of Surgery, Toronto General Hospital, 200 Elizabeth Street, Toronto, Ontario, M5G 2C4, Canada; 9 Cancer Care Ontario, 620 University Avenue, Toronto, Ontario, M5G 2L7, Canada

## Abstract

**Background:**

It is unclear if all persons in Ontario have equal access to colonoscopy. This research was designed to describe long-term trends in the use of colonoscopy by primary care physicians (PCPs) in Ontario, and to determine whether PCP characteristics influence the use of colonoscopy.

**Methods:**

We conducted a population-based retrospective study of PCPs in Ontario between the years 1996-2005. Using administrative data we identified a screen-eligible group of patients aged 50-74 years in Ontario. These patients were linked to the PCP who provided the most continuous care to them during each year. We determined the use of any colonoscopy among these patients. We calculated the rate of colonoscopy for each PCP as the number of patients undergoing colonoscopies per 100 screen eligible patients. Negative binomial regression was used to identify factors associated with the rate of colonoscopy, using generalized estimating equations to account for clustering of patients within PCPs.

**Results:**

Between 7,955 and 8,419 PCPs in Ontario per year (median age 43 years) had at least 10 eligible patients in their practices. The use of colonoscopy by PCPs increased sharply in Ontario during the study period, from a median rate of 1.51 [inter quartile range (IQR) 0.57-2.62] per 100 screen eligible patients in 1996 to 4.71 (IQR 2.70-7.53) in 2005. There was substantial variation between PCPs in their use of colonoscopy. PCPs who were Canadian medical graduates and with more years of experience were more likely to use colonoscopy after adjusting for their patient characteristics. PCPs were more likely to use colonoscopy if their patient populations were predominantly women, older, had more illnesses, and if their patients resided in less marginalized neighborhoods (lower unemployment, fewer immigrants, higher income, higher education, and higher English/French fluency).

**Conclusions:**

There is substantial variation in the use of colonoscopy by PCPs, and this variation has increased as the overall use of colonoscopy increased over time. PCPs whose patients were more marginalized were less likely to use colonoscopy, suggesting that there are inequities in access.

## Background

Colonoscopy - endoscopic evaluation of the lower gastrointestinal tract - is a test frequently performed for the diagnosis and therapy of colonic conditions, as well as for colorectal cancer (CRC) screening, and has been used increasingly in Canada over the past 15 years. Variations in the use of other health services [1] including cancer screening-such as breast cancer and cervical cancer screening--have been reported in Canada [2]. Greater use of health services may not apply uniformly to all patients. For example, when access to magnetic resonance imaging (MRI) increased in Ontario, it was preferentially used by patients with higher socioeconomic status [3]. Colonoscopy is a limited resource that has recently increased 3-fold in use from 1992 to 2001 [4]. Primary Care Physician (PCP) recommendation is one of the strongest determinants of whether patients have a colorectal cancer screening test [5-7]. Referrals may be influenced by a number of factors in addition to medical need, such as PCPs' perceptions of colonoscopy's risks and benefits, and the preferences of their patients.

The objective of this study was to describe long-term trends in the use of colonoscopy by PCPs in Ontario for their patients, and to determine whether PCP characteristics, and the characteristics of their patients, influence access to colonoscopy in Ontario in an era of increasing colonoscopy use.

## Methods

We conducted a population-based retrospective study of PCPs in Ontario between the years 1996-2005, to measure the variation in PCP use of colonoscopy among their patients and to evaluate factors affecting the use of colonoscopy over time.

### Data Sources

We used six data sources: (1) The Ontario Cancer Registry (OCR), a registry of all Ontario residents newly diagnosed with cancer or who died from cancer since 1964, estimated to be 95% complete [8]; (2) The Canadian Institute for Health Information-Discharge Abstract Database (CIHI-DAD), which contains information on all discharges from acute care facilities for residents of Ontario dating from 1988 including clinical information on diagnoses, procedures and discharge status; (3) The Ontario Health Insurance Plan (OHIP) database, which contains information on claims for physicians' services and all medical procedures to the Ontario Ministry of Health and Long-Term care made by fee-for-service physicians, community based laboratories and radiology facilities; (4) The Registered Persons Data Base (RPDB), which contains demographic information for all residents eligible for health care in Ontario; (5) The ICES Physician Database (IPDB), which contains information about physician demographics, specialty training and practice location in Ontario; and (6) The 2001 Canadian Census files, which contain aggregated data that describe general demographic information of the Canadian population at the census tract level.

### Identification of screen-eligible patients

We were interested in studying colonoscopy use among patients in whom colorectal cancer screening might be considered. Patients with a previous or new diagnosis of colorectal cancer, or those in whom cancer is strongly suspected, are very likely to undergo colonoscopy; variation between PCPs in the use of colonoscopy for these patients is likely to vary little. Colonoscopy performed for diagnosis of suspected colorectal cancer, known lower gastrointestinal disease or acute presentation is not highly discretionary. However, most colonoscopies are done in patients at low risk of having colorectal cancer.

Residents of Ontario aged 50-74 years who were eligible for OHIP benefits for each calendar year from 1996-2005, had no prior diagnosis of CRC, inflammatory bowel disease (IBD), Ulcerative colitis, Crohn's disease, or colonic polyps, and who received care from a PCP in Ontario, were considered as screen-eligible patients. Patients who had colon or rectal surgery at any time prior to January 1 of each calendar year, or who had a colonoscopy for any reason in the previous 4 years were excluded. Patients residing in regions where physicians do not bill directly for services were not included (approximately 4.5% of the Ontario population). The exclusion criteria and corresponding diagnosis and service codes are shown in Additional File 1: Appendix 1.

### Linking patients to a PCP

Each patient with two or more encounters with a general practitioner/family physician (GP/FP) in Ontario was assigned to a PCP based on health services received during each calendar year from 1996-2005. For each potential patient we identified all outpatient OHIP service codes claimed by a GP/FP and calculated the number of visits made to GP/FPs. For each calendar year, we assigned each patient to a single PCP. For those patients who had visits to more than one GP/FP over the year, we used the continuity of care (COC) measure [9,10] to determine the PCP most responsible for providing continuous care.

We used the method for estimating COC described by Bice and Boxerman [11] which can be estimated for patients who have had at least two visits to a GP/FP over the year. All visits to either a usual provider or a referred provider are attributed to a single provider (the referring provider is considered to be the 'usual provider' for the purpose of estimating the COC measure). A score of one represents perfect COC.

### Identification of colonoscopy

Based on OHIP billing codes, we identified all colonoscopies (Z555) performed on patients linked to a PCP for each calendar year from 1996-2005. For individuals who had more than one colonoscopy during the study period, only the first colonoscopy was considered. We were also interested in differentiating colonoscopy performed for the investigation or work up of a known or highly probable CRC from those performed on a more discretionary basis, such as CRC screening. It was not possible to differentiate screening colonoscopies using our data sources. Therefore, we developed an algorithm to identify colonoscopies--in retrospect--that were very likely to have been performed for CRC screening, or for another relatively discretionary indication. A proportion of these colonoscopies would even considered "unnecessary" by some endoscopists. A discretionary colonoscopy was defined as a colonoscopy procedure performed on a patient of screen-eligible age, not performed during an inpatient stay, and not associated with diagnosis of CRC at the time of colonoscopy or within a 3 year period following the colonoscopy (Additional File 1: Appendix 2). Because most referrals to specialists in this time period originated from PCPs [12], we attributed performance of the colonoscopy to the patient's PCP although colonoscopy was in general performed by a specialist physician who had seen the patient at the request of a PCP.

### Identification of physician and screen-eligible patient characteristics

We collected information from the IPDB on PCP characteristics such as age, sex, number of years in practice, education (Canadian training vs. International Medical Graduate), and rurality of the area of practice. Information on characteristics of patients treated by PCPs was derived both at the patient level (age, sex and comorbidity) and at the neighborhood level (income, rurality, level of education, employment, immigration status and language fluency). We determined co-morbid conditions for subjects using the Charlson comorbidity score [13,14] based on hospital discharges up to four year prior to their entry into the study. Those with no hospital admissions were assigned a Charlson score of zero. Patient characteristics were aggregated at the PCP level to characterize the patient population for each PCP.

Patient neighborhood information at the level of the census dissemination area (DA, the smallest geographic area for which census data are made available by Statistics Canada) was used to specify covariates at an ecological level. Each patient's DA of residence was identified based on their postal code and then linked to the Statistics Canada postal code conversion file (PCCF). The neighborhood information was gathered from the 2001 Canadian census based on each patient's DA. For each patient we determined the percentage of patients who resided in the lowest income quintile (Q1) neighborhood. For each patient, we also determined the percentage of the population aged 20 and older with less than a high school diploma, the percentage that was a visible minority (persons other than Aboriginal peoples, who are non-white), the percentage of the population aged 25 and older that was unemployed, the percentage of the population living in a rural area and the percentage of the neighborhood population that did not speak an official language (English or French). Once these neighborhood-level variables were defined for each patient, they were then aggregated at the PCP level using a weighted average by taking the size of the DA into account to define the average characteristics of the patients within his or her practice.

### Statistical Analysis

The unit of analysis was the PCP, and all patient-level information was aggregated at the PCP level. PCPs' use of colonoscopy and discretionary colonoscopy was measured as a rate per 100 screen-eligible patients during each calendar year. To avoid unstable estimates based on small denominators, and to ensure we only included PCPs who routinely refer patients for colonoscopy, only PCPs who contributed more than two years of data and who were linked to at least 10 patients per year were included in analyses. We used negative binomial regression [15] to explore the relationship between the rate of colonoscopy and PCP characteristics. We modeled the number of colonoscopies for each PCP as a count, with the logarithm of the eligible population of each PCP as the offset variable, including other physician and patient-level characteristics as covariates. Since the data were right-skewed and the variance was greater than the mean, the negative binomial model provided an improved fit to the data and accounted for over-dispersion better than a Poisson regression model [16,17]. The median rate of colonoscopy was calculated for each year stratified by different characteristics. We selected categories to make the number of subjects approximately equal. The categories were all defined a priori so as not to select optimal cut-points to maximize the study findings. Variation in colonoscopy use between PCPs was tested using F-test for equality of variance. In the multivariate analyses, a generalized estimating equations (GEE) approach [18] was used to account for repeated measures from each PCP. The trend over time was examined by modeling year as a continuous explanatory variable. Interactions between the trend over time and each of the patient characteristics were tested. A piecewise regression model was fitted to test for sudden changes in trend in any specific year. The only year with a significant instantaneous change was 1999. Due to the high degree of collinearity, the following variables were excluded in the final multivariate model: age of PCP, rural location of PCP practice and proportion of visible minorities. All tests were two-sided and all analyses were performed using SAS software system version 9.1. Adjusted rate ratios (RR) and 95% confidence intervals (CIs) are reported.

### Ethics

This study was approved by the research ethics board of Sunnybrook Health Science Centre, Toronto.

## Results

Of 13,098 PCPs in practice at any time during the study period, 1,969 were in practice less than 2 years or had fewer than 10 screen-eligible patients in any year of the study, yielding a total of 11,129 PCPs. Each year from 1996 to 2005 there were between 7,955 and 8,419 eligible PCPs in Ontario with a median age of 43 years. The distribution of PCPs and their screen eligible patients' characteristics for each calendar year is summarized in Table 1.

**Table 1:** Characteristics of Primary Care Physicians (PCPs) and screen eligible patients 1996-2005

Characteristics	1996	1997	1998	1999	2000	2001	2002	2003	2004	2005
	No (%)	No (%)	No (%)	No (%)	No (%)	No (%)	No (%)	No (%)	No (%)	No (%)
**Primary Care Physician characteristics**										
Number of physicians	7955	8008	7960	8013	8077	8133	8174	8273	8365	8419
Male	5642 (70.9)	5628 (70.3)	5553 (69.8)	5521 (68.9)	5519 (68.3)	5509 (67.7)	5482 (67.1)	5500 (66.5)	5519 (66.0)	5512 (65.5)
Age group										
< 45	3906 (49.1)	3806 (47.5)	3646 (45.8)	3512 (43.8)	3411 (42.2)	3274 (40.3)	3172 (38.8)	3108 (37.6)	3016 (36.1)	2886 (34.28)
>= 45	4049 (50.9)	4202 (52.5)	4314 (54.2)	4501 (56.2)	4666 (57.8)	4859 (59.7)	5002 (61.2)	5165 (62.4)	5349 (63.9)	5533 (65.7)
Years in practice										
< 20	4277 (53.8)	4191 (52.3)	4055 (50.9)	3940 (49.2)	3851 (47.7)	3732 (45.9)	3639 (44.5)	3561 (43.0)	3483 (41.6)	3333 (39.6)
>= 20	3656 (45.9)	3787 (47.4)	3894 (48.9)	4062 (50.7)	4213 (52.2)	4390 (53.9)	4527 (55.4)	4702 (56.8)	4870 (58.2)	5071 (60.2)
Canadian Medical Graduates	6087 (76.5)	6155 (76.9)	6156 (77.3)	6218 (77.6)	6280 (77.8)	6330 (77.8)	6358 (77.8)	6437 (77.8)	6484 (77.5)	6482 (77.0)
Rural area of practice	882 (11.1)	872 (10.9)	860 (10.8)	882 (11.0)	898 (11.1)	808 (9.9)	826 (10.1)	829 (10.0)	855 (10.2)	835 (9.9)
Screen eligible patient count										
< 200	4665 (58.6)	4628 (57.7)	4437 (55.7)	4373 (54.6)	4322 (53.5)	4321 (53.1)	4298 (52.6)	4369 (52.8)	4370 (52.2)	4337 (51.5)
>= 200	3290 (41.4)	3380 (42.2)	3523 (44.3)	3640 (45.4)	3755 (46.5)	3812 (46.9)	3876 (47.4)	3904 (47.2)	3995 (47.8)	4082 (48.5)
**Screen eligible patient characteristics, Averaged at the level of each Primary Care Physician**										
Median age										
50-59	3068 (38.6)	3468 (43.3)	3748 (47.1)	3958 (49.4)	4323 (53.5)	4578 (56.3)	4742 (58.0)	4932 (59.6)	5084 (60.8)	5352 (63.6)
60-61	2193 (27.6)	2152 (26.9)	2061 (25.9)	2078 (25.9)	2024 (25.1)	1988 (24.4)	2056 (25.2)	2052 (24.8)	2083 (24.9)	1965 (23.3)
> 61	2694 (33.9)	2388 (29.8)	2151 (27.0)	1977 (24.7)	1730 (21.4)	1567 (19.3)	1376 (16.8)	1289 (15.6)	1198 (14.3)	1102 (13.1)
% male pts										
< 35	2028 (25.5)	2056 (25.7)	2068 (25.9)	2104 (26.3)	2159 (26.7)	2182 (26.8)	2177 (26.6)	2189 (26.5)	2188 (26.2)	2180 (25.9)
35-50	3192 (40.1)	3049 (38.1)	2974 (37.4)	2829 (35.3)	2854 (35.3)	2777 (34.1)	2810 (34.4)	2813 (34.0)	2841 (33.9)	2813 (33.4)
> 50	2735 (34.4)	2903 (36.3)	2918 (36.7)	3080 (38.4)	3064 (37.9)	3174 (39.0)	3187 (38.9)	3271 (39.5)	3336 (39.9)	3426 (40.7)
% of with any comorbidities										
< 10	1717 (21.6)	1814 (22.7)	1851 (23.3)	1911 (23.9)	1990 (24.6)	2093 (25.7)	2288 (27.9)	2524 (30.5)	2794 (33.4)	3087 (36.7)
10-15	2715 (34.1)	2799 (34.9)	2861 (35.9)	2923 (36.5)	3016 (37.3)	3019 (37.1)	3020 (36.9)	3119 (37.7)	3236 (38.7)	3282 (38.9)
> 15	3523 (44.3)	3395 (42.4)	3248 (40.8)	3179 (39.7)	3071 (38.0)	3021 (37.1)	2866 (35.1)	2630 (31.8)	2335 (27.9)	2050 (24.4)
**Ecological variables**										
% of low income quintile										
< 10	2524 (31.7)	2560 (31.9)	2594 (32.6)	2460 (30.7)	2542 (31.5)	2565 (31.5)	2626 (32.1)	2724 (32.9)	2082 (24.9)	2152 (25.6)
10-19	2453 (30.8)	2531 (31.6)	2539 (31.9)	2517 (31.4)	2574 (31.9)	2638 (32.4)	2685 (32.9)	2715 (32.8)	3013 (36.0)	3044 (36.2)
>= 20	2978 (37.4)	2917 (36.4)	2827 (35.5)	3036 (37.9)	2961 (36.7)	2930 (36.0)	2863 (35.0)	2834 (34.3)	3270 (39.1)	3223 (38.3)
% of rural pts										
< 1	2802 (35.2)	2766 (34.5)	2678 (33.6)	2862 (35.7)	2816 (34.9)	2732 (33.6)	2682 (32.8)	2695 (32.6)	2858 (34.2)	2831 (33.6)
1-3	2118 (26.6)	2120 (26.5)	2140 (26.9)	2207 (27.5)	27.8)	2274 (27.9)	2285 (27.9)	2315 (27.9)	2299 (27.5)	2355 (27.9)
> 3	3035 (38.2)	3122 (38.9)	3142 (39.5)	2944 (36.7)	3014 (37.3)	3127 (38.5)	3207 (39.2)	3263 (39.4)	3208 (38.4)	3233 (38.4)
% with less than high school education										
20-24	1918 (24.1)	2001 (24.9)	1947 (24.5)	2025 (25.3)	1983 (24.6)	1992 (24.5)	1995 (24.4)	2024 (24.5)	2068 (24.7)	2089 (24.8)
>= 25	3534 (44.4)	3484 (43.5)	3440 (43.2)	3356 (41.9)	3393 (42.0)	3328 (40.9)	3318 (40.6)	3311 (40.0)	3323 (39.7)	3340 (39.7)
Missing	236 (2.9)	232 (2.9)	211 (2.7)	237 (2.9)	226 (2.8)	295 (3.6)	325 (3.9)	327 (3.9)	379 (4.5)	370 (4.4)
% of visible minority										
< 10	2605 (32.8)	2571 (32.1)	2555 (32.1)	2509 (31.3)	2584 (31.9)	2610 (32.1)	2634 (32.2)	2619 (31.4)	2665 (31.9)	2716 (32.3)
10-24	2713 (34.1)	2788 (34.8)	2771 (34.8)	2813 (35.1)	2817 (34.9)	2752 (33.8)	2756 (33.7)	2842 (34.4)	2782 (33.3)	2785 (33.1)
>= 25	2401 (30.2)	2417 (30.2)	2423 (30.4)	2454 (30.6)	2450 (30.3)	2476 (30.4)	2459 (30.1)	2485 (30.0)	2539 (30.4)	2548 (30.3)
Missing	236 (2.9)	232 (2.9)	211 (2.7)	237 (2.9)	226 (2.8)	295 (3.6)	325 (3.9)	327 (3.9)	379 (4.5)	370 (4.4)
% of non-immigrants										
< 55	1780 (22.4)	1754 (21.9)	1765 (22.2)	1770 (22.1)	1757 (21.8)	1812 (22.3)	1827 (22.4)	1804 (21.8)	1896 (22.7)	1872 (22.2)
55-74	2884 (36.3)	2943 (36.8)	2860 (35.9)	2902 (36.2)	2891 (35.8)	2869 (35.3)	2878 (35.2)	2931 (35.4)	2862 (34.2)	2870 (34.1)
>= 75	3291 (41.4)	3311 (41.4)	3335 (41.9)	3341 (41.7)	3429 (42.5)	3452 (42.4)	3469 (42.4)	3538 (42.8)	3607 (43.1)	3677 (43.7)
% in labour force										
< 65	2804 (35.3)	2699 (33.7)	2568 (32.3)	2477 (30.9)	2470 (30.6)	2353 (28.9)	2277 (27.9)	2247 (27.2)	2176 (26.0)	2121 (25.2)
65-69	3039 (38.2)	3059 (38.2)	3092 (38.8)	3099 (38.7)	3057 (37.9)	3067 (37.7)	3066 (37.5)	3077 (37.2)	3118 (37.3)	3132 (37.2)
>= 70	1876 (23.6)	2018 (25.2)	2089 (26.2)	2200 (27.5)	2324 (28.8)	2418 (29.7)	2506 (30.7)	2622 (31.7)	2692 (32.2)	2796 (33.2)
Missing	236 (2.9)	232 (2.9)	211 (2.7)	237 (2.9)	226 (2.8)	295 (3.6)	325 (3.9)	327 (3.9)	379 (4.5)	370 (4.4)
% cannot speak English/French										
< 2	2632 (33.1)	2614 (32.6)	2666 (33.5)	2647 (33.0)	2682 (33.2)	2724 (33.5)	2725 (33.3)	2761 (33.4)	2755 (32.9)	2804 (33.3)
2-3	2972 (37.4)	3017 (37.7)	2948 (37.0)	2989 (37.3)	3040 (37.6)	2992 (36.8)	3033 (37.1)	3069 (37.1)	3091 (36.9)	3092 (36.7)
> 3	2115 (26.6)	2145 (26.8)	2135 (26.8)	2140 (26.7)	2129 (26.4)	2122 (26.1)	2091 (25.6)	2116 (25.6)	2140 (25.6)	2153 (25.6)
Missing	236 (2.9)	232 (2.9)	211 (2.7)	237 (2.9)	226 (2.8)	295 (3.6)	325 (3.9)	327 (3.9)	379 (4.5)	370 (4.4)

Table 2 presents the PCP rate of any colonoscopy and discretionary colonoscopy among their patients for each year from 1996 to 2005. The rates were expressed as the number of colonoscopies per 100 eligible patients in a PCP's practice. The median PCP rate of colonoscopy was 1.51 in 1996 and 4.71 in 2005. There was wide variation between PCPs in their use of colonoscopy, and this variation increased over the 10 year period from an inter-quartile range of 2.05 in 1996 to 4.83 in 2005 (P < 0.001).

**Table 2:** Trends in the use of colonoscopy by Ontario PCPs from 1996-2005

Year	No: of PCPs	No: of screen eligible patients assigned to PCP	No: of colonoscopies	Any colonoscopy		Discretionary colonoscopy	
				**Median** **rate^+^**	**25th-75**^th^ **percentile**	**Median** **rate^+^**	**25th-75^th ^** **percentile**

1996	7,955	1,512,592	24,540	1.51	0.57 - 2.62	1.26	0.38 - 2.31
1997	8,008	1,557,533	27,719	1.65	0.68 - 2.82	1.43	0.51 - 2.54
1998	7,960	1,598,434	32,478	1.86	0.86 - 3.23	1.62	0.67 - 2.89
1999	8,013	1,641,273	38,953	2.21	1.08 - 3.66	1.97	0.89 - 3.37
2000	8,077	1,680,316	46,862	2.56	1.32 - 4.25	2.33	1.11 - 3.92
2001	8,133	1,713,337	56,899	3.03	1.59 - 4.87	2.76	1.37 - 4.55
2002	8,174	1,733,178	65,576	3.4	1.85 - 5.56	3.12	1.61 - 5.22
2003	8,273	1,747,927	70,725	3.67	1.93 - 5.88	3.38	1.68 - 5.56
2004	8,365	1,781,850	83,072	4.08	2.25 - 6.63	3.77	2.00 - 6.25
2005	8,419	1,806,977	98,464	4.71	2.70 - 7.53	4.39	2.41 - 7.20

Note: PCP = Primary Care Physician

+ Per 100 screen eligible patients in practice

**Figure 1:**
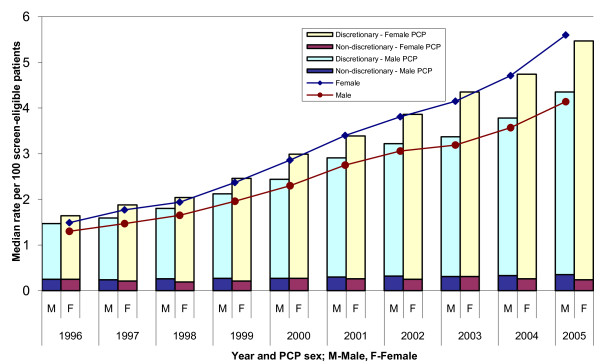
**Use of colonoscopy by sex of primary care physician**. Note: The line graph shows the median rate of discretionary colonoscopy among male and female PCPs, who contributed to all 10 years of the study period (71% of the PCPs in 1996).

Figure 1 shows the use of colonoscopy according to the sex of the PCP from 1996 to 2005. The median rate of colonoscopy was significantly lower for male physicians than for women. Over time, as the overall rates of colonoscopy increased, the difference between the groups became larger. The increased use of colonoscopy over time appeared to be entirely due to an increase in the use of discretionary colonoscopy; the rate of non-discretionary colonoscopy did not change substantially over the 10 year time period.

**Figure 2:**
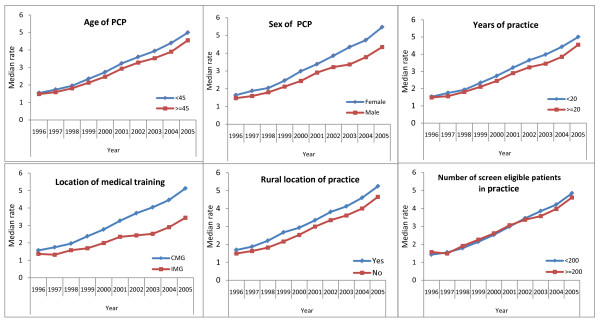
**Rate of colonoscopy by PCP characteristics**. Note: PCP = Primary Care Physician, CMG - Canadian Medical Graduates, IMG - International Medical Graduates.

**Figure 3:**
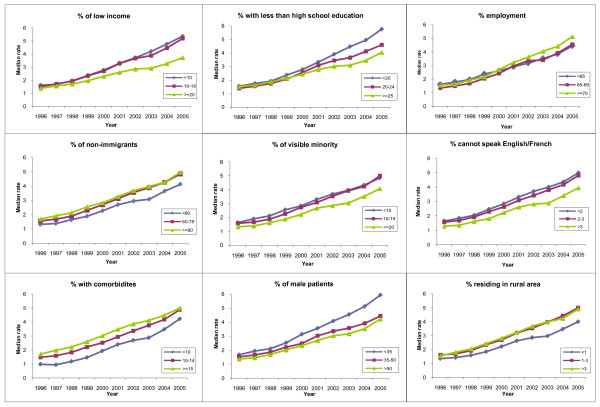
**Rate of colonoscopy according to measures of neighbourhood marginalization and other characteristics of screen-eligible patients**. Note: The interaction between the trend and each characteristics was statistically significant (P < .001).

The rate of colonoscopy according to PCP characteristics is shown in Figure 2. There was an increase in the use of colonoscopy among PCPs who were less than 45 years of age, Canadian medical graduates and those who practice in a rural area of location. Figure 3 illustrates trends in the use of colonoscopy by PCPs according to their average practice characteristics.

Table 3 displays the results of the multivariate analysis of physician and patient factors associated with PCP rate of colonoscopy. Use of colonoscopy was influenced by the PCPs' demographic and practice characteristics. PCPs who graduated from a medical school outside Canada (RR = 0.78, 95% CI 0.77-0.79) and who had less than 20 years in practice (RR = 0.96, 95% CI 0.94-0.97), were significantly less likely to use colonoscopy after adjusting for their patient characteristics. Patient characteristics including age, sex, comorbidities, and ecologic patient-related variables such as neighborhood income, education, unemployment, percent of non-immigrants and language fluency were all significantly associated with a PCP's rate of colonoscopy after adjusting for their physician characteristics. PCPs with a lower proportion of screen-eligible patients with comorbid diseases were less likely to use colonoscopy for their patients. PCPs with practices that included less marginalized patients (higher income, higher level of education, employment, non-immigrants, more English/French fluency) were more likely to use colonoscopy than other PCPs. Some of these associations are very small, although statistically significant. We tested whether each category is significantly different from the reference category and the corresponding p-values are presented in the Table. The overall significance of each of the covariates was checked using likelihood ratio chi-square statistics. All the covariates included in the model except PCP sex were statistically significant (P < 0.01) in determining the PCP rate of colonoscopy. We also tested the statistical significance between different categories of each covariate and found that it is significant for % male patients (< 35 vs. 35-50, p < 0.001), % co-morbidity (< 10 vs. 10-15, p < 0.01), and % with less than high school education (< 20 vs. 20-24, p < 0.001), % of non-immigrants (< 60 vs. 60-79, p < 0.05), % of labour force (< 65 vs. 65-69, p < 0.05) and % cannot speak English or French (< 2 vs. 2-3, p < 0.05). Interactions between the time trends and, sex and age of both PCP and patients, were explored but not found to be statistically significant.

**Table 3:** Associations between colonoscopy and PCP characteristics - Estimates from multivariate Analyses

Parameters		Risk Ratio	95% CI	P-value
Year		1.35	1.30-1.40	< 0.0001

**Physician characteristics**				

Sex	Female	1.03	0.99-1.06	0.1354
	Male	1.00	-	-

Years of experience	< 20	0.95	0.94-0.97	< 0.0001
	>= 20	1.00	-	-

Education	IMG	0.78	0.77-0.79	< 0.0001
	CMG	1.00	-	-

**Practice characteristics**				

**Individual level**				

% of male patients	< 35	1.18	1.14-1.22	< 0.0001
	35-50	1.02	1.01-1.04	0.0011
	> 50	1.00	-	-

Median age	50-59	0.96	0.94-0.98	< 0.0001
	60-61	0.97	0.96-0.99	0.0002
	>= 62	1.00	-	-

% co-morbidity	< 10	0.71	0.70-0.72	< 0.0001
	10-15	0.85	0.84-0.86	< 0.0001
	> 15	1.00	-	-

**Ecological level**				

% low income	< 10	1.14	1.12-1.15	< 0.0001
	10-19	1.13	1.11-1.14	< 0.0001
	>= 20	1.00	-	-

% of lives in rural location	< 1	1.01	0.98-1.02	0.5212
	1-3	1.03	1.02-1.05	< 0.0001
	> 3	1.00	-	-

% with less than high school education	< 20	1.28	1.26-1.30	< 0.0001
	20-24	1.08	1.06-1.09	< 0.0001
	>= 25	1.00	-	-

% of non-immigrants	< 60	0.88	0.85-0.90	0.0011
	60-79	0.95	0.94-0.97	< 0.0001
	>= 80	1.00	-	-

% of labour force	< 65	0.97	0.96-0.99	0.0005
	65-69	0.94	0.93-0.96	< 0.0001
	>= 70	1.00	-	-

% cannot speak English or French	< 2	0.92	0.89-0.94	< 0.0001
	2-3	0.96	0.94-0.98	< 0.0001
	> 3	1.00	-	-

Note: RR - rate ratio, CI - Confidence Interval, CMG - Canadian Medical Graduates, IMG - International Medical Graduates.

* Categories were selected to include approximately equal numbers of subjects.

## Discussion

The use of colonoscopy has increased in Ontario. However, the increase in colonoscopy use has not been uniform for all patients. We found increasing social disparities in use of colonoscopy as it increased over a decade. We made several important observations about the use of colonoscopy by PCPs and the factors that are associated with its use. First, there was a 4-fold increase in use of colonoscopy over the 10-year study period, which was almost entirely explained by an increase in discretionary colonoscopy. Second, there was a substantial variation in the use of colonoscopy at the level of the PCP. This variation increased as the overall use of colonoscopy increased. Third, utilization increased disproportionately for PCPs whose patients resided in less marginalized neighborhoods (higher levels of income and education). Fourth, certain physician groups, including Canadian medical graduates and who had more years of experience, were more likely to refer their patients for colonoscopy than other PCPs. Finally, patient characteristics, including age, sex, presence of comorbidities, neighborhood income, area of residence, neighborhood level of education, neighborhood rate of unemployment, neighborhood level of non-immigrants and neighborhood knowledge of English or French were significantly associated with PCP rate of colonoscopy. These factors could not be explained by PCP characteristics such as age and sex.

The PCPs included in our study were representative of Ontario PCPs. The age-sex distribution was similar to the Ontario family physician/general practitioner data published by National Physician survey in 2004 [19]. The use of colonoscopy in our study was similar to other published population-based studies in Ontario [20,21]. Our study supports previous research showing that patient socioeconomic status [22,23], and racial and ethnic factors [24] play a significant role in CRC screening. The higher rate of colonoscopy among less marginalized patient groups may be due to wealthier patients demanding a higher intensity of health services, or because patients in more marginalized neighborhoods may not access colonoscopy due to difficulties in transportation and time off work. This study was performed in Ontario, Canada, which has a public single-payer health care system. Our results may not be generalizable to other jurisdictions where the organization, financing and delivery of health services differ.

Our study has limitations. First, because of the cross-classified structure of the data, patients may not be completely nested within physicians. To address this limitation, we incorporated a measure of continuity of care to ensure that the PCP assigned to a patient was most responsible for providing continuous care over a period of time. Second, using the definition of COC, patients were required to have at least 2 visits/year for entry to the study. Hence, we might have missed a small proportion of healthier patients who might have only 1 or no visits/year. However, such asymptomatic patients would have been less likely to have been sent for colonoscopy during the 10 years compared to those with more visits. Third, although we sought to distinguish discretionary colonoscopy from diagnostic colonoscopy, it is impossible to reliably establish the indication for a colonoscopy using Ontario claims data. Fourth, some potential confounders that are associated with the likelihood of colonoscopy were not measured in our study, such as a family history of CRC. Fifth, we used ecological level measures as a proxy for patient level data on neighborhood income, level of education, unemployment, immigration status, and language; and some misclassification may have occurred. However, our findings are consistent with previous studies that show socioeconomic status and racial and ethnic differences are associated with health service utilization including CRC screening [22]. Ecologic studies can provide population level insights that can be extrapolated to individuals [25,26]. Sixth, it is possible that consultant endoscopists vary in their likelihood to perform a colonoscopy. Our study did not differentiate between patients who did not receive a colonoscopy because they were not referred from those who did not receive a colonoscopy because of the judgment of the consultant endoscopist. The limitations of our study design did not substantially affect our ability to accomplish our principal research objectives.

## Conclusions

In conclusion, our population-based study of Ontario PCPs and their patients demonstrates that there is a wide variation in the use of colonoscopy among PCPs, and this variation increased as the overall use of colonoscopy increased over a decade. As access to colonoscopy increased over time, there were increasing social disparities in its use. Decision makers should be aware that access to colonoscopy is strongly associated with socioeconomic status of patients. Further work is necessary to determine how variation in use of colonoscopy affects CRC and other health outcomes.

## Competing interests

The authors declare that they have no competing interests.

## Authors' contributions

BJJ was responsible for the data collection, analyses and interpretation of data, drafting the manuscript, the process of seeking funding. NNB made significant contributions to interpretations of data and also took part in editing the manuscript. RM and RS were the resource persons for statistical analysis, assisted with interpretation of the data and helped in editing the manuscript. LDG is a primary care physician who assisted in editing the manuscript. DRU was responsible for the conception and design of study, manuscript. All of the authors read and approved the final manuscript.

## Pre-publication history

The pre-publication history for this paper can be accessed here:

http://www.biomedcentral.com/1471-230X/11/102/prepub

## Supplementary Material

Additional file 1 **APPENDICIES**. Appendix 1 Exclusion criteria and corresponding diagnosis or billing codes for identifying screen eligible. Population. Appendix 2 Exclusion criteria for identifying discretionary colonoscopies (colonoscopies likely to be done for screening purposes). Click here for file
