# Supplementary material for: Social disparities in the use of colonoscopy by primary care physicians in Ontario
Source: BMC Gastroenterol. 2011 Sep 28;11:102. doi: 10.1186/1471-230X-11-102 (PMC3206464; doi:10.1186/1471-230X-11-102)
Supplement: Additional file 1 — APPENDICIES. Appendix 1 Exclusion criteria and corresponding diagnosis or billing codes for identifying screen eligible. Population. Appendix 2 Exclusion criteria for identifying discretionary colonoscopies (colonoscopies likely to be done for screening purposes). [file 1471-230X-11-102-S1.DOCX]

**APPENDICIES**

**Appendix 1 Exclusion criteria and corresponding diagnosis or billing codes for identifying screen eligible population**

| **Exclusion criteria** | **Data source** | **Corresponding diagnostic or billing codes** |
| --- | --- | --- |
| Previous diagnosis of CRC prior to January 1^st^ of each calendar year | OCR | ICD-9 codes 153.0-153.4, 153.6-154.1  ICD-10 codes C18.0-C20 |
| Previous diagnosis of IBD/Ulcerative colitis/Crohn’s disease prior to January1^st^ of each calendar year | CIHI-DAD | ICD -9 codes 556, 556.0-556.9,  ICD-9 code 555, 555.0-555.9  ICD-10 codes K50.0-50.9, K51.0-51.9 |
| Subjects with polyps removed prior to Januray1^st^ of each calendar year | OHIP | Z570, E719, Z571, E720, Z764, Z765, E685 in addition to the code Z555 on same day |
| Colonoscopy in the previous 4 years | OHIP | All Z555 codes |
| Colon or rectal resections prior to January 1^st^ of each calendar year | OHIP,  CIHI-DAD | S162, S166, S167, S169, S172, S171, S168, S170, S173, S174, S188, S177, S213, S214, S215, S216, S217  CCP procedure codes 57.5x, 57.6x, 60.4x, 60.5x  ICD-10 procedure codes 1.NM.87x, 1.NM.89x, 1.NM.91x, 1.NQ.87x, 1.NQ.89x, 1.NQ.90x |
| Residing in the area where physicians do not bill directly for services | RPDB | Kingston SEAMO – South East LHIN |

**Appendix 2 Exclusion criteria for identifying discretionary colonoscopies (colonoscopies likely to be done for screening purposes)**

| **Exclusion criterion** | **Data source** | **Corresponding diagnosis or procedure codes** |
| --- | --- | --- |
| Inpatient colonoscopies | OHIP  CIHI-DAD | OHIP fee codes that occur during a hospitalization in CIHI  (i.e. admission date <= date of colonoscopy <= discharge date) |
| Colonoscopies where a diagnosis of colorectal cancer in OCR occurred within 3 years of the colonoscopy | OCR | ICD-9 codes 153.0-153.4, 153.6-154.1  ICD-10 codes C18.0-C20 |
| Colonoscopies on patients at high risk for receiving a diagnosis of CRC |  | Exclude patients with previous colonoscopy in the previous 4 years (index colonoscopy may be done for surveillance rather than screening purposes), diagnosis of prior CRC (elevated risk for subsequent CRC), or inflammatory bowel disease (elevated risk of developing CRC in patients with ulcerative colitis) |
